# The Relationship of Neuropsychological Variables to Driving Status Following Holistic Neurorehabilitation

**DOI:** 10.3389/fneur.2014.00056

**Published:** 2014-04-23

**Authors:** Ramaswamy Kavitha Perumparaichallai, Kristi L. Husk, Stephen M. Myles, Pamela S. Klonoff

**Affiliations:** ^1^Center for Transitional NeuroRehabilitation, Barrow Neurological Institute, St. Joseph’s Hospital and Medical Center, Phoenix, AZ, USA

**Keywords:** neurorehabilitation, cognitive outcome, driving, brain injury, neuropsychological functioning

## Abstract

**Objective:** The main objectives of the present study were to evaluate the cognitive and driving outcomes of a holistic neurorehabilitation program and to examine the relationship between the neuropsychological variables of attention, speed of information processing, and visuospatial functioning and driving outcomes.

**Methods:** One hundred and twenty-eight individuals with heterogeneous neurological etiologies who participated in a holistic neurorehabilitation program. Holistic neurorehabilitation consisted of therapies focusing on physical, cognitive, language, emotional, and interpersonal functioning, including training in compensatory strategies. Neuropsychological testing was administered at admission and prior to starting driving or program discharge. Subtests of processing speed, working memory, and perceptual reasoning from the Wechsler Adult Intelligence Scale-III and Trail Making Test were included.

**Results:** At the time of discharge, 54% of the individuals returned to driving. Statistical analyses revealed that at the time of discharge: the sample as a group made significant improvements on cognitive measures included in the study; the driving and non-driving groups differed significantly on aspects of processing speed, attention, abstract reasoning, working memory, and visuospatial functions. Further, at the time of admission, the driving group performed significantly better than the non-driving group on several neuropsychological measures.

**Conclusion:** Cognitive functions of attention, working memory, visual-motor coordination, motor and mental speed, and visual scanning significantly contribute to predicting driving status of individuals after neurorehabilitation. Holistic neurorehabilitation facilitates recovery and helps individuals to gain functional independence after brain injury.

## Introduction

Return to driving is one of the primary goals of neurorehabilitation after brain injury, which is associated with enhanced quality of life (1–5). In an outcome study related to holistic multidisciplinary neurorehabilitation, Leon-Carrion et al. (6) reported the benefits of neurorehabilitation within the context of improving driving skills. Their study predicted that more than 70% of survivors of severe traumatic brain injury (TBI) could return to driving. Earlier studies from our center (3, 7), emphasized the role of neurorehabilitation programs in preparing individuals after brain injury to enhance their cognitive skills as well as emotional functioning; these are essential for driving. Klonoff et al. (3) explored the relationship between performance on cognitive retraining tasks and driving outcome. Driving ability was associated with better performance on cognitive retraining tasks that addressed cognitive functions of processing speed, focused attention, visual scanning, and memory (3). A significant association between meta-cognitive skills (e.g., use of compensatory strategies, organizational, and procedural skills) and emotional functioning in the form of maintaining therapeutic relationships, with clearance to drive was also reported.

Depending upon the characteristics of the sample included in the previous studies, there is variability in the percentage of individuals who returned to driving. In an epidemiological study of social reintegration of individuals who had suffered severe brain injury and coma, 32% of individuals returned to driving (8). Fifty percent of participants with moderate to severe TBI went back to driving within 5 years and most of those within 1 year of injury (4).

Driving requires a dynamic interaction of physical, emotional, and cognitive skills, including perceptual and higher executive functions (9, 10). A neurological insult to the brain affects the harmonious interplay of these functions. A large number of studies have reported the adverse impact of neurocognitive impairments on driving abilities, including: visuoperception (11, 12), attention/concentration (11–17), speed of information processing (11, 13, 15–17), memory (11, 17), executive systems (11–13, 16, 17), emotional regulation (2), and personality factors (18).

Several variables have been identified as contributing to return to driving; however, research indicates that the severity of brain injury and neuropsychological functioning are most predictive of an individual’s ability to drive after brain injury (2, 6, 8, 19). Among the neuropsychological variables, attention and processing speed have demonstrated higher predictive validity (18). Increasingly, practitioners in neuropsychological rehabilitation centers are called upon to assess individuals’ fitness to drive after brain injury. Heterogeneous neuropsychological profiles, variable recovery patterns, and lack of consistent clinical guidelines and regulations create complexities in evaluating individuals’ capacity to drive post-brain injury (10, 20). However, not all individuals who returned to driving post-brain injury underwent a formal evaluation of their ability to drive (9, 21), which increased accident risk (6).

Our center, a milieu-oriented holistic multidisciplinary neurorehabilitation center, uses data from multiple sources (e.g., performance on cognitive retraining tasks and neuropsychological tests, various measures of visuo-perceptual functioning from Occupational Therapy, and emotional stability) to assess individuals’ fitness to return to driving. Depending upon individuals’ performance on the above-mentioned variables, a physician referral is made for an Adaptive Driving Evaluation before the individual is formally released to drive. This tends to be an expensive procedure. In order to increase success rates on adaptive driving evaluations, it is important to understand the association between the above-mentioned variables and driving outcomes. The main objectives of the present study were: (i) to evaluate the cognitive as well as driving outcomes in a milieu-oriented holistic neurorehabilitation program and (ii) to examine the relationship between the performance on tests of attention, speed of information processing, and visuospatial functioning and driving outcomes following milieu-oriented holistic neurorehabilitation.

## Methodology

### Sample

The sample consisted of 128 individuals with brain injury who participated in milieu-oriented holistic neurorehabilitation therapies at the Center for Transitional NeuroRehabilitation (CTN). Neuropsychological test scores of individuals who participated between the years of 2000 and 2010 were collected retrospectively from records. The inclusion criteria of this study were: (1) participation in at least one or more of the following programs: Home Independence, Work Re-Entry, or School Re-Entry Programs; (2) individuals were driving before their brain injury; however, they were not driving at the time of admission, secondary to their brain injuries; (3) ages 16 or older; (4) a minimum of 8 weeks of milieu-oriented holistic neurorehabilitation; and (5) individuals who underwent both admission and follow-up neuropsychological evaluations.

Table 1 contains demographic and clinical data. At admission, the mean age of sample was 34.7 years, ranging from age 16 to 63 years. The average level of education was 13.96 years. Of this sample, 59.4% were male and 89.1% were right-hand dominant. The majority of the sample consisted of Caucasians (84.4%). The remainder of the sample consisted of 7.8% Hispanic, 3.1% Asian, 0.8% African American, and 3.9% other ethnic groups.

**Table 1:** **Demographic and clinical data**.

Variables (*n* = 128)	Mean (SD)	Range	Median
Age at admission (in years)	34.7 (13.75)	16–63	33.5
Education (in years) mean (SD)	13.96 (2.63)	8–20	14.0
Treatment duration (in months) mean (SD)	10.27 (5.12)	2–33	9.75
Injury-to-admission duration (in months)	10.42 (23.89)	0.5–230	4.0

	**Frequency (%)**

Injury-to-admission duration
≤2 years (%)	118 (92.2)	
>2 years (%)	10 (7.8)	
Handedness right/left	114/14 (89.1/10.9)	
Gender male/female	76/52 (59.4/40.6)	
Ethnicity
Caucasian	108 (84.4)	
African American	1 (0.8)	
Hispanic	10 (7.8)	
Asian	4 (3.1)	
Others	5 (3.9)	
Etiology
TBI	75 (58.6)	
CVA	36 (28.1)	
Anoxia	7 (5.5)	
Tumor	8 (6.3)	
Infection	2 (1.6)	
Neurorehabilitation program
Home independence	20 (15.6)	
School re-entry	13 (10.2)	
Work re-entry	74 (57.8)	
School + work re-entry	21 (16.4)	
Driving status at discharge
Driving	69 (53.9)	
Not driving	59 (46.1)	

In terms of brain injury etiologies, 58.6% of the sample sustained TBI. Over one-fourth (28.1%) of this sample sustained cerebrovascular accidents (CVA), which included hemorrhagic and ischemic stroke, aneurysmal rupture, and arteriovenous malformations (AVM), while the remainder of the sample were: tumors (6.3%); anoxic brain injuries (5.5%); and neuro-infectious disorders (1.6%). In the present study, the majority (at least 90%) of the sample sustained moderate to severe brain injuries. Severity was estimated retrospectively using the Glasgow Coma Scale (GCS) score, duration of posttraumatic amnesia (when available), and other neurological deficits (e.g., neurosurgical evacuation of intracranial hematomas, clear and persisting neurological signs such as hemiparesis or ataxia). The mean injury to admission interval was 10.42 months (range = 2 weeks to 230 months). The majority of the sample (92.2%) was <2 years post-injury, with 7.8% of individuals >2 years post-injury. The mean duration of treatment was 10.27 months (range = 2–33 months). With regard to the neurorehabilitation program, 57.8% of individuals were in the Work Re-Entry program; 16.4% were in the School and Work Re-Entry programs; 10.2% were in the School Re-Entry program; and 15.6% were in the Home Independence program.

### Intervention

Intensive milieu-oriented holistic neurorehabilitation consisted of therapies focusing on physical, cognitive, emotional, and interpersonal functioning, including training in compensatory strategies. The philosophy of the CTN program is based upon the principles of awareness, acceptance, and realism (22). Therapy focused on increasing individuals’ awareness of their limitations, skill building through the use of compensatory strategies, and cognitive retraining to remediate specific deficit areas. Participants received therapies in Neuropsychology, Speech-Language Pathology, Occupational Therapy, Physical Therapy, and Recreational Therapy. Most of the participants also received services in the areas of Physiatry, Psychiatry, and Dietary. The majority of the sample attended clinic based therapies generally four days per week, six hours per day. As they improved, therapies transitioned into community settings (e.g., home, work, or school).

Description of the holistic milieu-oriented neurorehabilitation programs is beyond the scope of this paper; for more details, see Klonoff (22). Briefly, the general goals of the Home Independence program focus on improving individuals’ functionality and quality of life (e.g., independence in basic self-care and activities of daily living; unsupervised time; independence with transportation needs; and involvement in leisure activities). The objectives of the Work and School Re-Entry programs are to facilitate successful transition to paid or volunteer work and school, respectively. During the transition period to work and/or school, the individuals obtained support from the therapists in: job skills/study skill straining and compensatory strategies. Therapists also served as liaisons with employers/teachers to facilitate accommodations.

### Neuropsychological evaluation

All of the participants included in the present study underwent a comprehensive neuropsychological evaluation at admission and a repeat neuropsychological evaluation at the conclusion of their neurorehabilitation program or before returning to driving. For the purposes of the present study, the scores on the measures of attention, speed of information processing, and visuospatial functioning were extracted retrospectively from their medical records. Subtests of digit span (DS), arithmetic (ART), letter number sequencing (LNS), symbol search (SS), digit symbol coding (DSC), block design (BD), and matrix reasoning (MR) from the Wechsler Adult Intelligence Scale-Third Edition (WAIS-III) and Trail Making Test (TMT) Parts A and B were included.

## Results

In the present study, 69 (54%) individuals returned to driving and 59 (46%) did not return to driving following their participation in the neurorehabilitation program. Table 2 summarizes a comparison of demographic data between driving and non-driving groups. The driving and non-driving groups did not differ significantly on the demographic variables of age and gender. The groups differed significantly on the variables of education, treatment duration, and injury-to-admission interval. Attempts to control the variability related to injury-to-admission interval were not successful, secondary to a smaller proportion of individuals (*n* = 10) who were above 2 years post-injury. Data were analyzed using SPSS version 18 for Windows. All tests were two-tailed. The results were provided in three sections, as follows: (i) overall cognitive outcome; (ii) differences in cognitive outcome between driving and non-driving groups; and (iii) predictability of driving status.

**Table 2:** **Demographic variables and driving status**.

Variables	Driving (*n* = 69)	Non-driving (*n* = 59)
Age in years mean (SD)	36.68 (14.0)	32.37 (13.18)
Education in years* mean (SD)	14.54 (2.73)	13.29 (2.37)
Gender** male/female (%)	39/30 (57/43)	37/22 (63/37)
Injury-to-admit in months* mean (SD)	5.70 (7.51)	15.93 (33.57)
Treatment duration in months* mean (SD)	9.2 (3.98)	11.53 (5.98)

*The driving and non-driving groups were significantly different using *t*-test, *p* < 0.05; **χ^2^ = 0.51; *p* = 0.48.

### Overall cognitive outcome

**Figure 1:**
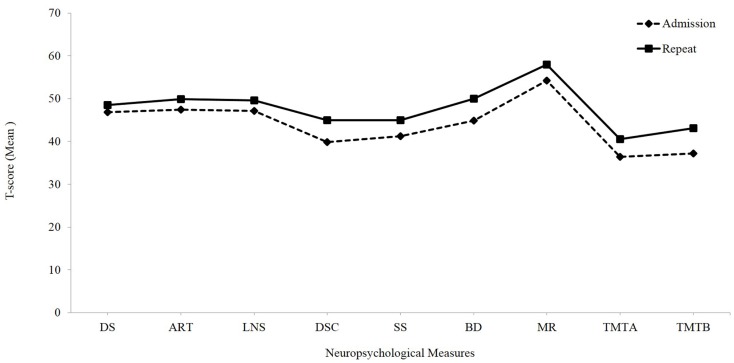
**Overall cognitive outcome: mean *t*-scores of neuropsychological measures at admission and repeat evaluations independent of the driving status**. DS, digit span; ART, arithmetic; LNS, letter number sequencing; SS, symbol search; BD, block design; MR, matrix reasoning; TMT A, Trail Making Test, Part A; TMT B, Trail Making Test, Part B.

Paired *t*-tests conducted independent of the driving status between the admission and repeat evaluations revealed significant improvements from admission to repeat testing on all of the cognitive measures included in the study (*p*-value after adjusting for multiple comparisons; Bonferroni correction was set at 0.006) (see Table 3; Figure 1).

**Table 3:** **Paired *t*-test between the admission and repeat neuropsychological test scores**.

Test variables	Admission *t*-scores mean (SD)	Repeat *t*-scores mean (SD)	*p*-Value
Digit span (DS)	46.83 (8.67)	48.48 (9.26)	0.006*
Arithmetic (ART)	47.36 (10.06)	49.90 (10.12)	0.0001*
Letter number sequencing (LNS)	47.13 (10.00)	49.61 (9.83)	0.0001*
Digit symbol coding (DSC)	39.78 (10.28)	44.98 (10.84)	0.0001*
Symbol search (SS)	41.22 (10.01)	44.99 (10.86)	0.0001*
Block design (BD)	44.86 (8.34)	49.50 (8.21)	0.0001*
Matrix reasoning (MR)	54.21 (10.21)	57.94 (8.09)	0.0001*
Trail Making Test (TMT)
Part A	36.41 (13.96)	40.45 (14.18)	0.0001*
Part B	37.22 (15.23)	43.11 (14.30)	0.0001*

*p-Value after adjusting for multiple comparisons; Bonferroni correction was set at 0.006.

### Differences in cognitive outcome between the driving and non-driving groups

**Figure 2:**
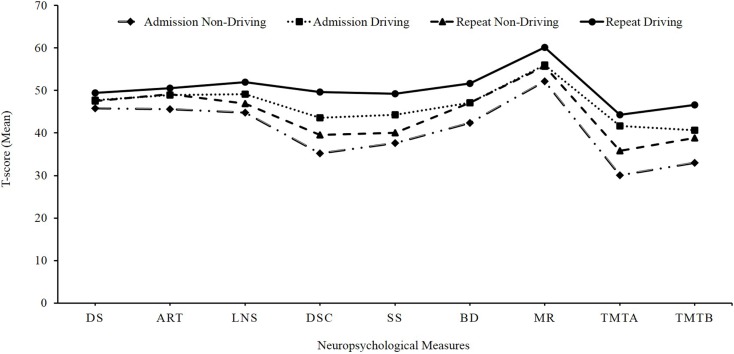
**Differences in cognitive outcome between driving and non-driving groups: mean *t*-scores of neuropsychological measures at admission and repeat evaluations**. DS, digit span; ART, arithmetic; LNS, letter number sequencing; SS, symbol search; BD, block design; MR, matrix reasoning; TMT A, Trail Making Test, Part A; TMT B, Trail Making Test, Part B.

Table 4 contains the results of a Repeated Measures Analysis of Variance (RMANOVA) using admission and repeat neuropsychological test scores with driving status as the between subjects variable. The analysis revealed that the driving and non-driving groups differed significantly on the cognitive functions of: verbal working memory LNS (*F* = 8.64; df = 1, 124; *p* < 0.004); speed of information processing DSC (*F* = 35.40; df = 1, 123; *p* < 0.0001) and SS (*F* = 23.94; df = 1, 123; *p* < 0.0001); visuoconstructional ability BD (*F* = 10.87; df = 1, 111; *p* < 0.001); sustained attention TMT Part A (*F* = 21.53; df = 1, 121; *p* < 0.0001); and divided attention TMT Part B (*F* = 10.93; df = 1, 119; *p* < 0.001) (*p*-value after adjusting for multiple comparisons; Bonferroni correction was set at 0.006). In addition, the cognitive measure of visual abstract reasoning (MR) trended towards significance (*F* = 6.88; df = 1, 104; *p* < 0.01) (see Figure 2).

**Table 4:** **Differences in cognitive outcome between driving and non-driving groups using repeated measures ANOVA**.

Variables	Admission mean (SD)		Repeat mean (SD)		*p*-Value
	Non-driving	Driving	Non-driving	Driving	
DS	45.76 (8.46)	47.74 (8.80)	47.46 (9.48)	49.36 (9.05)	0.19
ART	45.56 (10.76)	48.94 (9.19)	49.10 (11.10)	50.60 (9.19)	0.15
LNS	44.81 (10.29)	49.10 (9.38)	46.86 (10.01)	51.96 (9.10)	0.004*
DSC	35.21 (9.51)	43.62 (9.34)	39.47 (10.14)	49.60 (9.16)	0.0001*
SS	37.56 (9.95)	44.29 (9.05)	40.00 (9.65)	49.18 (10.07)	0.0001*
BD	42.44 (8.72)	47.07 (7.38)	47.11 (8.40)	51.68 (7.45)	0.001*
MR	52.24 (11.55)	56.04 (8.49)	55.67 (8.09)	60.05 (7.57)	0.01
TMT
Part A	30.11 (14.77)	41.67 (10.80)	35.79 (14.85)	44.34 (12.42)	0.0001*
Part B	32.96 (15.53)	40.66 (14.19)	38.76 (14.97)	46.61 (12.81)	0.001*

*p-Value after adjusting for multiple comparisons (Bonferroni correction was set at 0.006); DS, digit span; ART, arithmetic; LNS, letter number sequencing; SS, symbol search; DSC, digit symbol coding; BD, block design; MR, matrix reasoning; and TMT, Trail Making Test.

Exploratory *t*-tests were conducted to investigate the differences in cognitive functioning between the driving and non-driving groups before and after the treatment (see Table 5). On both admission and repeat testing, the driving group performed significantly better than the non-driving group on DSC (*t* = −4.94, df = 124, *p* = 0.001; *t* = −5.37, df = 125, *p* = 0.001), SS (*t* = −3.92, df = 124, *p* = 0.001; *t* = −5.37, df = 125, *p* = 0.001), BD (*t* = −3.73, df = 125, *p* = 0.001;*t* = −3.23, df = 112, *p* = 0.002), TMT Part A (*t* = −4.95, df = 122, *p* = 0.001;*t* = −3.26, df = 125, *p* = 0.001), and TMT Part B (*t* = −2.84, df = 119, *p* = 0.005;*t* = −3.47, df = 124, *p* = 0.001). Furthermore, the driving group’s performance was significantly better on the measures of LNS (*t* = −3.2, df = 126, *p* = 0.002) and MR (*t* = −2.89, df = 104, *p* = 0.005) on repeat testing. Of note, during the admission testing, on the other variables of LNS (*t* = −2.45, df = 124, *p* = 0.02) and MR (*t* = −2.20, df = 126, *p* = 0.03), the driving group performed better than the non-driving group, which trended towards significance (*p*-value after adjusting for multiple comparisons was 0.006).

**Table 5:** **Independent samples *t*-test between driving and non-driving groups at admission and repeat testing**.

Variables	Admission testing mean (SD)		*p*-Values	Repeat testing mean (SD)		*p*-values
	Driving	Non-driving		Driving	Non-driving	
DS	47.74 (8.80)	45.76 (8.46)	0.20	49.36 (9.05)	47.46 (9.48)	0.25
ART	48.88 (9.06)	45.56 (10.76)	0.06	50.60 (9.19)	49.10 (11.10)	0.41
LNS	49.10 (9.38)	44.81 (10.29)	0.02	51.93 (9.04)	46.41 (10.52)	0.002*
DSC	43.57 (9.29)	35.21 (9.51)	0.001*	49.60 (9.16)	39.17 (10.12)	0.001*
SS	44.19 (9.03)	37.56 (9.95)	0.001*	49.18 (10.07)	39.66 (9.84)	0.001*
BD	48.10 (8.16)	42.59 (8.47)	0.001*	52.13 (8.18)	47.11 (8.40)	0.002*
MR	56.09 (8.20)	52.22 (11.59)	0.03	60.05 (7.57)	55.67 (8.09)	0.005*
TMT
Part A	41.50 (10.81)	30.11 (14.77)	0.001*	43.93 (12.79)	35.80 (15.32)	0.001*
Part B	40.66 (14.19)	32.96 (15.53)	0.005*	46.61 (12.81)	37.90 (15.34)	0.001*

*p-Value after adjusting for multiple comparisons (Bonferroni correction was set at 0.006); DS, digit span; ART, arithmetic; LNS, letter number sequencing; SS, symbol search; DSC, digit symbol coding; BD, block design; MR, matrix reasoning; and TMT, Trail Making Test.

### Predictability of driving status

Clinically, neuropsychological test scores are among key factors included in assessing an individual’s ability to drive. A logistic regression analysis was conducted to evaluate the ability of cognitive measures to predict driving outcome. In the present study, only the results of repeat neuropsychological testing were included in the logistic regression. Per the logistic regression analysis, the Hosmer–Lemeshow test suggested that the model has a good fit: χ^2^ = 3.72, df = 8, *p* = 0.882. Furthermore, the model explained 51.7% of the variance, suggesting a moderately strong relationship between prediction and grouping. The Wald criterion demonstrated that the cognitive measures of attention, working memory, and processing speed predicted significantly, as follows: ART (Wald = 6.022, df = 1, OR = 0.899, *p* = 0.014); LNS (Wald = 6.21, df = 1, OR = 1.12, *p* = 0.013); and DSC (Wald = 9.887, df = 1, OR = 1.125, *p* = 0.002). Of note, collinearity diagnostics ruled out the threat of multi-collinearity among the independent variables to the model’s validity.

## Discussion

The present study examined the cognitive and driving outcomes of individuals with brain injuries following a milieu-oriented holistic neurorehabilitation treatment program. The present study supports the notion that milieu-oriented holistic neurorehabilitation enhances an individual’s fundamental cognitive functions pertinent to return to driving after a moderate to severe brain injury.

### Overall cognitive outcome

Cognitive outcome was examined using paired *t*-tests between admission and repeat neuropsychological test scores independent of driving status. The results indicated that irrespective of their driving status at the time of discharge, all participants made significant progress on cognitive functions of attention, speed of information processing, visuo-constructional ability, and executive functions (e.g., working memory, abstract reasoning, and multi-tasking) following neurorehabilitation. The results also demonstrated that individuals with moderate to severe brain injuries, regardless of their injury-to-admission interval, had the potential to improve on basic cognitive measures essential to enhance their functional independence. Several studies have documented the benefits of cognitive rehabilitation in increasing the functional independence of individuals with a TBI or stroke (23). They emphasized the need for randomized controlled studies to evaluate the efficacy of cognitive neurorehabilitation. Improvements demonstrated in the present study could be partly related to spontaneous recovery. One of the limitations of the present study is that the efficacy of the neurorehabilitation treatment could not be estimated, secondary to lack of a control group.

### Driving outcome

The ability to return to driving following brain injury is one of the important milestones of neurorehabilitation towards achieving functional independence, community re-integration, and quality of life (2, 5, 24). In the present study, 69 (54%) individuals returned to driving following neurorehabilitation. This is higher than the earlier driving outcome studies, which reported a range of 32–51% of their samples with moderate to severe brain injuries returned to driving (4, 8). Participants in the present study underwent milieu-oriented holistic multidisciplinary neurorehabilitation, which focused on improving meta-cognitive and executive functioning (e.g., awareness training) and compensation training in addition to basic cognitive remediation. Earlier studies implicated the role of meta-cognitive skills (e.g., executive functioning and emotional regulation) in the return to safe driving (12, 17). However, the results of the present study are different from earlier outcome studies from holistic multidisciplinary programs, which noted that at least 70% of their samples returned to driving or possessed abilities to return to driving post-brain injury. The participants of these studies were comparable to the present sample in terms of severity of brain injury and treatment (6, 25). We speculate that the difference in driving outcomes between our study and previous neurorehabilitation outcome studies may be a reflection of the shorter time from injury to the assessment of return to driving, as these individuals may continue to make neurological gains. For example, the follow-up study by Klonoff et al. (25) included individuals 1–7 years post-treatment, which suggests that some individuals may need more time to re-acquire driving skills. Long-term follow-up studies could help us understand the extended recovery patterns.

### Differences in cognitive functioning between driving and non-driving groups

One of the main objectives of the present study was to understand the relationship between cognitive outcomes and driving status. A RMANOVA indicated that driving and non-driving groups differed significantly on cognitive measures of processing speed and working memory following neurorehabilitation. Intuitively, one would expect that the driving group would outperform the non-driving group on several cognitive measures included in the present study. Prior studies have reported that the individuals who returned to driving performed better on several cognitive measures including, processing speed and working memory than those who did not return to driving (11, 15, 17).

It is noteworthy that exploratory *t*-tests between driving and non-driving groups revealed that the driving group performed significantly better on several aspects of cognition at baseline. An earlier study from our center (3) reported that individuals who returned to driving differed significantly on treatment variables, performance on cognitive retraining tasks, as well as process variables such as working alliance and compensation use. With a better understanding of baseline assessment and recovery patterns, it may be possible to predict the ability to drive much earlier in the treatment process. This would help individuals and their families estimate short-term and long-term transportation needs, which is one of the important aspects of caregiving.

### Predictability of driving status

A regression analysis indicated that higher scores on DSC, ART, and LNS significantly predicted driving status. The DSC subtest pertains to visual-motor coordination, motor and mental speed, visual working memory, and visual scanning. The ART and LNS subtests reflect attention, mental control and alertness, reasoning, and working memory. The role of these functions in driving has been well documented in the literature (6, 9). Previous studies have reported the efficacy of cognitive measures, particularly those related to speed of information processing, visual scanning, and motor speed in predicting driving status (3, 26). DSC was one of the neuropsychological variables that contributed to discriminate 94.4% of competent drivers from non-competent drivers (26). Klonoff et al. (3) reported that better performance on a cognitive retraining task that addressed functions of speed of information processing, memory, motor speed, and visual scanning predicted driving status after neurorehabilitation.

It is interesting that the TMT (Parts A and B), which measure speed of attention, visual conceptual reasoning, and visuo-motor tracking, did not significantly predict driving status in the present study. The relationship between better performance on TMT and the ability to drive in older adults is well established (27, 28). This discrepancy in TMT’s predictability may be attributed to the differences in the sample characteristics (e.g., the age and neurological etiologies) between studies. The majority of the sample in the present study included young and middle-aged adults with acquired brain injuries, whereas the earlier studies included older adults with degenerative conditions.

Predicting driving status after brain injury has valuable clinical utility. Rehabilitation professionals are typically involved in determining a suitable timeframe for an individual after a brain injury to undergo an adaptive driving evaluation. The results from the present study can increase our understanding about the pattern of recovery and response to rehabilitation interventions within the context of returning to driving. This can assist rehabilitation professionals in providing psychoeducation for individuals and caregivers regarding the components of the return to driving process. In addition, this information can be used to increase the accuracy of clinical judgments related to choosing suitable individuals for driving evaluations. A comprehensive driving evaluation that includes an on-the road assessment (e.g., an adaptive driving evaluation) is the gold standard in driving assessments (1). Given the eagerness of the individuals to return to driving, stressors associated with brain injury and safety concerns of caregivers, it is imperative to accurately assess readiness to engage in an adaptive driving evaluation.

To conclude, the present study emphasized the importance of cognitive functions of attention, processing speed, and working memory in return to driving after brain injury. In the future, randomized control studies including larger sample sizes and a more homogeneous sample with a control group, as well as a comprehensive assessment of driving abilities would help healthcare professionals understand the role of neurorehabilitation in the return to driving process.

## Author Contributions

Designed the study: Pamela S. Klonoff, Kristi L. Husk, Ramaswamy Kavitha Perumparaichallai, Stephen M. Myles; Collected the data: Ramaswamy Kavitha Perumparaichallai, Kristi L. Husk; Analyzed the data: Ramaswamy Kavitha Perumparaichallai; Wrote the paper: Ramaswamy Kavitha Perumparaichallai, Kristi L. Husk, Pamela S. Klonoff.

## Conflict of Interest Statement

The authors declare that the research was conducted in the absence of any commercial or financial relationships that could be construed as a potential conflict of interest.
